# Autistic vs. Control Differences in MRI Scan Quality Across ABIDE-II Sites

**DOI:** 10.3390/diagnostics16101478

**Published:** 2026-05-13

**Authors:** João Pinheiro, Beatriz Afonso, Emanuel Cortesão de Seiça, Rita Gonçalves, Luís Ribeiro, Joana Reis

**Affiliations:** 1 Imaging Department, University of Algarve-Centre for Health Studies, 8005-139 Faro, Portugal; 2 Faculty of Psychology, University of Aveiro, 3800-193 Aveiro, Portugal; 3 Algarve Private Hospital Group, 8005-226 Faro, Portugal; 4 Faculty of Biomedical Sciences and Medicine, University of Algarve, 8005-139 Faro, Portugal; 5 Psychiatry Department, Hospital of Viana do Castelo, 4900-408 Viana do Castelo, Portugal

**Keywords:** autism spectrum disorder, mri data quality, multi-site neuroimaging, quality control bias, ABIDE-II

## Abstract

**Background:** Head motion and variability in scan quality remain major methodological challenges in autism neuroimaging. Large multi-site datasets such as ABIDE-II provide a unique opportunity to systematically quantify diagnostic differences in MRI data quality and assess the influence of site-level heterogeneity. **Methods:** Functional MRI Quality Assessment Protocol (QAP) metrics were combined with phenotypic data from ABIDE-II. Participants were classified as autistic (ASD) or typically developing (TD). Key quality metrics—including mean framewise displacement (mFD), proportion of volumes exceeding 0.20 mm (FD > 0.20), signal-to-noise ratio (SNR), and entropy focus criterion (EFC)—were analyzed alongside age, sex, IQ, and site. Group differences were evaluated using non-parametric tests and linear mixed-effects models with site as a random factor. Additional analyses examined site-level heterogeneity and the impact of quality-control (QC) thresholds on sample composition. **Results:** The final sample included 1277 participants (579 ASD; 698 TD) across 14 sites. ASD participants exhibited significantly greater head motion (median mFD = 0.101 vs. 0.081 mm; *p* < 1 × 10^−10^) and modest reductions in signal quality (lower SNR, higher EFC). Elevated motion in ASD was observed in 12 of 14 sites, although effect sizes varied substantially. Mixed-effects models confirmed that diagnosis remained a significant predictor of motion after adjusting for covariates. In contrast, signal-quality differences were small and largely explained by site effects. Simulated QC procedures disproportionately excluded ASD participants, with exclusion rates up to 31% compared to 18% in TD. **Conclusions:** ASD participants show consistently higher head motion, while signal-quality differences are minimal and largely site-driven. Standard QC procedures disproportionately exclude ASD individuals, highlighting the need for improved motion handling and more balanced quality-control strategies in multi-site studies.

## 1. Introduction

Autism spectrum disorder (ASD) is a neurodevelopmental condition characterized by differences in social communication, sensory processing, and patterns of restricted or repetitive behaviors, with substantial heterogeneity in cognitive and clinical profiles [1,2].

Understanding the neurobiological basis of this variability remains a central goal in autism research, and magnetic resonance imaging (MRI) has become a critical tool for examining brain structure, functional organization, and developmental trajectories in vivo [2,3]. However, the reliability of MRI-derived biomarkers depends fundamentally on the acquisition of high-quality data. Even subtle artifacts—particularly those caused by head motion—can distort estimates of cortical morphology, functional connectivity, and diffusion-based measures, leading to biased or spurious findings [4,5,6]. Because autistic individuals may experience heightened sensory sensitivity, anxiety, or difficulty maintaining stillness during scanning, they often exhibit increased in-scanner motion relative to typically developing peers [4,7,8]. Ensuring robust scan quality is therefore both scientifically essential and uniquely challenging in ASD research. High-quality imaging is a prerequisite for valid, reproducible neurobiological inference and for the development of clinically meaningful imaging markers in autism studies [9,10].

The present study systematically investigates differences in fMRI (Functional MRI) scan quality between ASD and TD participants across all ABIDE-II sites. Using functional quality assessment protocol (QAP) metrics merged with phenotypic data, we examine (i) global diagnostic differences in motion and signal characteristics, (ii) site-level modulation of ASD–TD differences, and (iii) diagnostic bias introduced by standard Quality-Control (QC) thresholds. Our findings provide methodologically critical insights into the interpretation, reporting, and reproducibility of autism neuroimaging results.

Autism neuroimaging research has been repeatedly challenged by head motion and scan-quality variability, both of which disproportionately affect studies involving neurodivergent populations. Even small differences in motion can systematically bias estimates of functional connectivity, cortical morphology, and diffusion-derived metrics, often producing artefactual group differences or masking true effects [10,11,12]. Because individuals with ASD may experience atypical sensory responses, anxiety, or reduced behavioral compliance during MRI acquisition, elevated motion has emerged as a central methodological concern in the field. Multiple studies have documented consistently greater in-scanner movement in ASD compared with typical TD controls, highlighting the robustness of this phenomenon across age groups and imaging modalities [4,7,8,13]. These motion-related artifacts have measurable consequences: they disproportionately degrade image quality, reduce usable data, and complicate interpretation of group-level neurobiological findings in ASD [11,14,15].

Large-scale initiatives such as the Autism Brain Imaging Data Exchange (ABIDE) and its second phase, ABIDE-II, provide unique opportunities to quantify and correct these confounds. ABIDE-II aggregates structural and functional MRI data from more than two dozen international sites, each employing distinct scanner hardware, acquisition parameters, participant recruitment strategies, and behavioral protocols [16]. This multi-site structure enables the identification of diagnostic patterns that generalize across settings while also revealing the extent to which site-level heterogeneity contributes to variation in scan quality. The availability of standardized image-quality metrics—derived from the MRI.

Quality Assessment Protocol (QAP) and informed by recent advances in automated QC frameworks [17,18,19]—further allows for systematic and reproducible assessment of scan integrity at scale.

Despite this progress, important methodological gaps remain. Prior work has demonstrated elevated motion in ASD relative to TD controls, but such analyses have often been limited to single-site datasets or have not directly modeled the interaction between diagnostic group and site-specific factors [4,8,13]. As a result, it is unclear whether ASD–TD differences in scan quality are consistent across imaging centers or whether they are amplified—or dampened—by site-level variations in equipment, sequence design, or participant management. Moreover, several studies have noted that QC procedures may differentially exclude ASD participants, thereby biasing final samples toward individuals with higher functioning levels, fewer sensory sensitivities, or greater behavioral compliance [14,15,19]. This potential sampling bias has significant implications: it may distort estimates of neural phenotypes in ASD, obscure true developmental trajectories, and reduce the generalizability of multisite findings.

Collectively, these challenges underscore the need for a systematic, multi-site investigation of MRI scan quality differences between ASD and TD groups using the full ABIDE-II dataset. By quantifying site heterogeneity, diagnostic effects, and the impact of QC thresholds on sample composition, such work can clarify whether observed differences reflect true group characteristics or methodological artefacts—and ultimately improve the interpretability and reproducibility of autism neuroimaging research.

Most existing studies control for motion but do not examine it as a primary outcome, rarely assess between-site heterogeneity, and do not evaluate the impact of QC procedures on sample composition. In addition, they typically do not leverage the full ABIDE-II dataset with standardized QAP metrics or apply comprehensive mixed-effects models, including diagnosis × age interactions. In contrast to prior work, the present study explicitly addresses these methodological gaps. First, we treat motion differences not merely as a nuisance variable but as a central outcome of interest, systematically quantifying diagnostic effects on multiple motion-related QAP metrics across the entire ABIDE-II sample. Second, we formally model between-site heterogeneity by incorporating site as a random effect in linear mixed-effects models and by conducting detailed site-wise profiling, allowing us to characterize how ASD–TD differences vary across acquisition environments. Third, we directly quantify the impact of common QC thresholds on diagnostic sample composition, demonstrating that standard motion-based exclusion procedures disproportionately remove autistic participants and thereby introduce systematic bias. Fourth, our analyses leverage the complete ABIDE-II functional MRI dataset together with its officially released QAP metrics, ensuring comprehensive and standardized measurement of scan quality across sites. Finally, we evaluate full mixed-effects models, including diagnosis x age interactions, to determine whether group differences vary across developmental stages. Together, these methodological advances provide a more rigorous and nuanced understanding of diagnostic differences in MRI scan quality than have been achieved in previous studies.

## 2. Materials and Methods

This study used publicly available fMRI quality metrics and phenotypic data from the ABIDE-II (Table 1). After merging QAP and phenotypic datasets using unique participants and site identifiers, the final sample for functional analyses included 1277 individuals from 14 imaging sites, comprising 579 ASD and 698 TD participants.

Phenotypic Variables

Diagnostic status was operationalized using the “DX_GROUP” variable from the ABIDE composite phenotypic dataset, where 1 = Autism Spectrum Disorder (ASD) and 2 = Typically Developing (TD). This binary classification served as the primary grouping factor in all analyses.

In addition to diagnostic group, several participant-level covariates known to influence MRI quality metrics were included. Age at scan (in years) was treated as a continuous predictor to capture developmental effects on head motion, tissue contrast, and other quality-related parameters. Sex was modeled as a categorical covariate (male/female), given its potential influence on both neurodevelopmental trajectories and MRI acquisition variability across sites. Full-scale IQ (FIQ) was incorporated as a continuous variable to control for cognitive variability between individuals and groups, minimizing confounding effects on quality metrics that may correlate with cognitive functioning.

All variables were extracted directly from the ABIDE composite phenotypic file, which harmonizes demographic, diagnostic, and cognitive data across contributing sites. Standard quality-control procedures were performed to ensure internal consistency, and participants with missing diagnostic status or incomplete covariate data were excluded listwise to maintain comparability across models.

QAP Metrics

fMRI scan quality was assessed using metrics derived from the ABIDE-II Quality Assessment Protocol (QAP) (Table 2). Motion-related metrics included mean framewise displacement (mFD) as a summary measure of overall head motion, along with the percentage and number of volumes exceeding 0.20 mm displacement, capturing the frequency and magnitude of excessive motion. Additional indicators of motion-related instability included outlier count and the median distance index (MDI).

To evaluate signal quality and spatial integrity, we analyzed signal-to-noise ratio (SNR), entropy focus criterion (EFC), spatial smoothness (FWHM), foreground-to-background energy ratio (FBER), and global correlation (GCORR). Together, these metrics provided a comprehensive characterization of motion, signal fidelity, and spatial consistency across scans. All metrics were analyzed on their original scales unless transformation was required for model diagnostics.

All metrics were retained in their original scales unless transformations were required during model diagnostics to improve normality or reduce heteroscedasticity.

**Statistical Analysis**

All statistical analyses were conducted to assess diagnostic group differences in MRI quality metrics while accounting for site heterogeneity, demographic covariates, and potential QC-related biases. Analyses proceeded in four stages: (1) group-level comparisons, (2) site-level profiling, (3) mixed-effects modeling, and (4) quality-exclusion simulations.

Group-Level Comparisons

Because several QAP metrics exhibited non-normal distributions, Mann–Whitney U tests were used to compare ASD and TD groups across the full dataset. Group differences were summarized using medians and interquartile ranges (IQRs) for each metric, including motion (mFD, PercentFD > 0.20, NumFD > 0.20, OutlierCount, MedianDistanceIndex) and signal/spatial metrics (SNR, EFC, FBER, FWHM, GCORR). Effect sizes were computed where appropriate to contextualize the magnitude of differences. To control for multiple comparisons across QAP metrics, false discovery rate (FDR) correction (Benjamini–Hochberg) was applied.

Site-Level Profiling and Heterogeneity Assessment

To assess site-level variability, all metrics were summarized within each diagnostic group:

- sample size;
- median and IQR;
- ASD–TD median differences;
- site-wise effect sizes.

Sites with fewer than 5 participants per diagnostic group were excluded from site-wise contrasts but retained in mixed-effects analyses to preserve hierarchical structure. Visualizations of site-level means were used to characterize between-site heterogeneity and identify sites contributing disproportionately to observed group differences.

Mixed-Effects Modeling

To evaluate diagnostic effects while accounting for multi-site data structure, linear mixed-effects models were fitted for each QAP metric. All models included age, sex, and full-scale IQ (FIQ) as covariates.

- Model 1—Random Intercept Model

$$QAP_{ij}=\beta_{0}+\beta_{1}DX_{ij}+\beta_{2}AGE_{ij}+\beta_{3}SEX_{ij}+\beta_{4}FIQ_{ij}+u_{0j}+\epsilon_{ij}$$

where $u_{0j}$ is a random intercept for site j.

This model estimated overall ASD–TD differences while adjusting for demographic covariates and site-level clustering.

- Model 2—Diagnosis × Age Interaction

For key metrics (notably mFD and SNR), interaction models were used to test whether ASD and TD groups differed in age-related trajectories:

$$QAP_{ij}=\beta_{0}+\beta_{1}DX_{ij}+\beta_{2}AGE_{ij}+\beta_{3}(DX\times AGE{)}_{ij}+\ldots$$

Predicted-value curves were derived for ASD and TD groups, allowing extraction of:

- group-specific slopes;
- difference in slopes (Δ slope);
- visual and statistical evaluation of non-parallel trajectories.

These analyses demonstrated (1) a modest diagnosis × age interaction for motion metrics and (2) a very small but detectable divergence in SNR trajectories. To assess the potential influence of ASD-only sites (i.e., sites without TD participants), a sensitivity analysis was performed by re-estimating all models after excluding these sites. This allowed evaluation of whether their inclusion biased global diagnostic effect estimates.

Site Interaction Model

For select analyses, a diagnosis x site interaction model was estimated to determine whether ASD–TD differences varied significantly across acquisition sites. This formally quantified the heterogeneity observed in descriptive visualizations.

Quality-Exclusion Bias Simulation

To evaluate whether common QC practices may introduce diagnostic bias, we simulated exclusion procedures using thresholds commonly applied in MRI preprocessing pipelines:

- mFD > 0.20 mm;
- PercentFD > 0.20 > 20%;
- and analogous thresholds for Outlier Count and SNR.
- For each rule, we computed:
- exclusion rates for ASD vs. TD participants;
- χ^2^ tests for group differences in exclusion proportions;
- changes in demographic distributions (age, FIQ, sex) before vs. after exclusion.

These simulations quantified the potential for QC filtering to disproportionately remove ASD participants and alter phenotypic composition, highlighting a source of systematic bias in autism neuroimaging studies.

The threshold of mFD > 0.20 mm was selected based on its widespread use in the neuroimaging literature as a standard criterion for identifying excessive head motion [6,11]. This cutoff reflects a balance between retaining sufficient data and minimizing motion-related artefacts and is commonly implemented in preprocessing pipelines and quality-control procedures. Accordingly, it provides a meaningful and ecologically valid benchmark for evaluating the potential impact of QC filtering on diagnostic sample composition.

Motion-matched and sensitivity analyses

To address the potential confounding effect of head motion on diagnostic differences in image quality, additional analyses were performed. First, a motion-matched subsample was constructed by matching individuals with ASD and TD controls on mFD. Matching was conducted using a nearest-neighbor approach without replacement, ensuring comparable distributions of head motion between groups while retaining as many participants as possible. All primary analyses were then repeated within this motion-matched subsample.

In addition, sensitivity analyses were conducted to evaluate the robustness of the findings to different motion thresholds. Specifically, the main models were re-estimated after excluding participants exceeding commonly used mFD thresholds (e.g., mFD > 0.20 mm and mFD > 0.15 mm). These analyses allowed assessment of whether the observed diagnostic effects persisted under increasingly stringent motion control criteria. Together, these complementary approaches were designed to disentangle the extent to which group differences in image quality reflect intrinsic behavioral characteristics versus residual motion-related artefacts.

## 3. Results

The final dataset comprised 1277 individuals from 16 ABIDE-II sites, including 579 ASD and 698 TD participants. Fourteen sites contributed data for both diagnostic groups; two sites (KUL_3, NYU_2) contributed only ASD participants and were excluded from site-wise ASD–TD contrasts but retained in global analyses and mixed-effects models.

### Global ASD vs. TD Differences in Motion and Scan Quality

Table 3 shows that autistic and typically developing participants differed only modestly in global signal and spatial quality metrics. Typically developing participants exhibited slightly higher SNR, indicating marginally cleaner BOLD signal given by the DVARS values, whereas the ASD group showed a higher Entropy Focus Criterion (EFC), a pattern consistent with increased blurring or reduced spatial focus often associated with motion-related instabilities. However, these effects, though statistically significant, were small in magnitude. The remaining spatial metrics, FBER, FWHM, GCORR, and the MDI, showed no significant ASD–TD differences, with group medians nearly overlapping. This pattern suggests that while motion contributes to subtle signal-level differences, the broader spatial integrity and global correlation structure of the scans are largely comparable across groups. Overall, autistic participants demonstrated mild reductions in signal quality but no widespread degradation in spatial characteristics, indicating that group contrasts in this domain are far less pronounced than those observed for motion.

**Figure 1:**
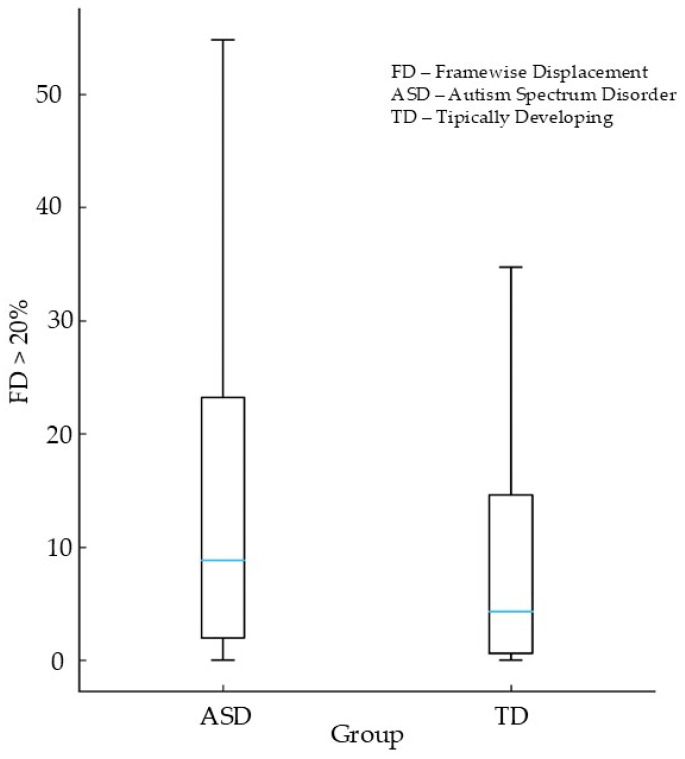
Percent FD > 0.20 mm (%) by Diagnostic Group.

Figure 1 illustrates the distribution of Percent FD > 0.20 mm across autistic and typically developing participants. The ASD group shows a clear rightward shift, with substantially higher percentages of volumes exceeding the 0.20 mm displacement threshold. This indicates that autistic participants not only move more on average but also experience more frequent high-displacement events throughout the scan. The distribution for TD participants is more left-skewed, with a larger proportion of individuals exhibiting very low rates of high-motion frames. The pronounced group separation in this figure visually reinforces the statistical findings reported in Table 3 that elevated motion is a robust and pervasive characteristic of the ASD sample—and highlights the potential for motion censoring or threshold-based QC procedures to disproportionately impact autistic participants.

**Figure 2:**
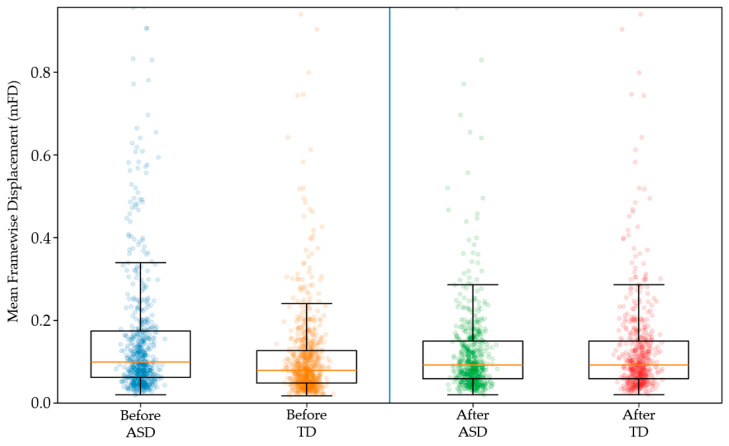
Distributioin of head motion before and after nearest-neighbor matiching.

ASD participants exhibited significantly higher head motion compared to TD controls. mFD distributions were determined for participants with ASD and TD controls before and after 1:1 nearest-neighbor matching without replacement (Figure 2). Matching was performed on log-transformed mFD using a caliper of 0.2 standard deviations and restricted to the region of common support. Before matching, ASD participants showed higher head motion than TD controls. After matching, the mFD distributions were closely aligned between groups, indicating successful balancing of motion and reducing motion-related confounding in subsequent analyses. Prior to matching, ASD participants exhibited substantially higher head motion. After restricting the sample to the overlapping range of mFD values, group distributions became more comparable, although some differences remained.

**Figure 3:**
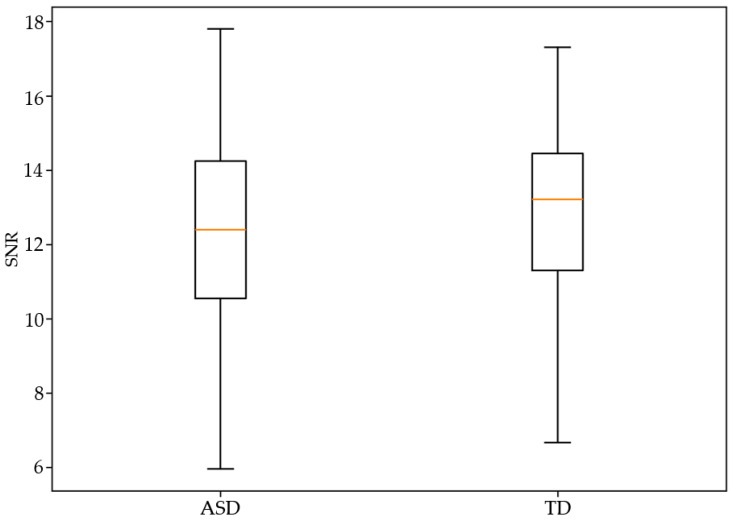
Image quality differences in the motion-matched sample.

Comparison of SNR between participants with ASD and TD controls after 1:1 nearest-neighbor matching on mean framewise displacement. Despite equivalent head motion between groups, differences in image quality metrics persisted, indicating that these effects are not solely attributable to motion-related artefacts (Figure 3).

Table 4 demonstrates that diagnostic differences in global signal and spatial MRI quality metrics were generally small and far less pronounced than those observed for motion. Typically developing participants showed modestly higher SNR, indicating slightly cleaner and more stable functional signal relative to autistic participants. Conversely, the ASD group exhibited significantly higher EFC, reflecting increased image entropy and reduced sharpness—an expected consequence of their elevated motion. However, beyond these two metrics, no other signal or spatial integrity measures differed significantly between groups. FBER, FWHM, GCORR, and the MDI showed overlapping distributions and negligible group differences. Collectively, these results indicate that although autistic participants display subtly reduced signal clarity, the overall spatial structure and global coherence of their scans remain broadly comparable to typically developing participants. Motion, rather than intrinsic image degradation, appears to be the dominant contributor to the small diagnostic disparities observed in signal-level quality.

To further characterize the role of acquisition site, we examined both within-site diagnostic differences and between-site variability in head motion and image quality metrics. Site-wise comparisons of mFD are summarized in Table 5, which presents diagnostic differences between participants with ASD and TD controls within each site. Across sites, ASD participants generally exhibited higher head motion than TD controls, although the magnitude and statistical significance of these differences varied considerably between sites.

**Figure 4:**
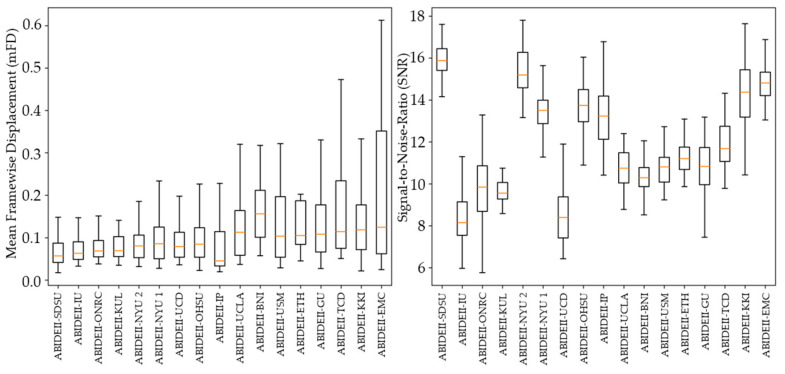
Inter-site variability in head motion and image quality. The left panel shows Head Motion by Site, summarized using mean framewise displacement (mFD), while the right panel shows Image Quality by Site, summarized using signal-to-noise ratio (SNR). Together, the panels illustrate substantial between-site variability in both participant motion and scan quality across ABIDE-II acquisition sites.

Complementing these analyses, Figure 4 illustrates the distribution of mFD and SNR across acquisition sites. Substantial variability was observed both between and within sites, with marked differences in central tendency and dispersion for both motion and image quality metrics. These findings highlight the heterogeneity inherent to multi-site datasets and support the inclusion of site as a random effect in subsequent mixed-effects models.

**Figure 5:**
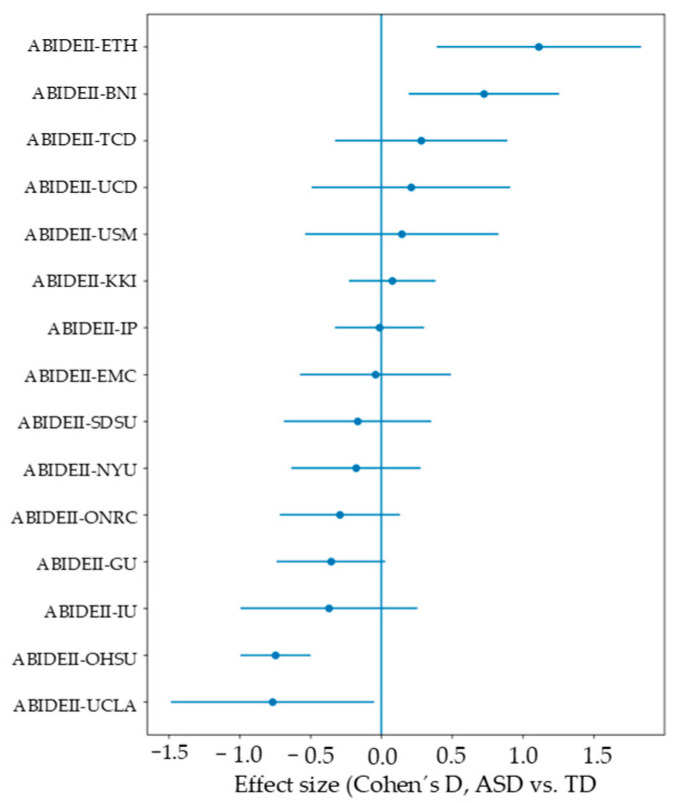
Forest plot of effect sizes (Cohen’s d) for SNR.

To further quantify this heterogeneity, site-specific effect sizes (Cohen’s d) for group differences in SNR were computed and are presented in Figure 5. Considerable variability was observed in both the magnitude and direction of effects across sites, with some sites showing higher SNR in ASD participants and others showing the opposite pattern. Confidence intervals were wide for several sites, particularly those with smaller sample sizes, indicating reduced precision of site-level estimates.

Taken together, these results demonstrate that both head motion and image quality metrics vary substantially across sites and that diagnostic effects are not uniform across acquisition contexts. This reinforces the importance of accounting for site-level variability in multi-site neuroimaging analyses.

**Figure 6:**
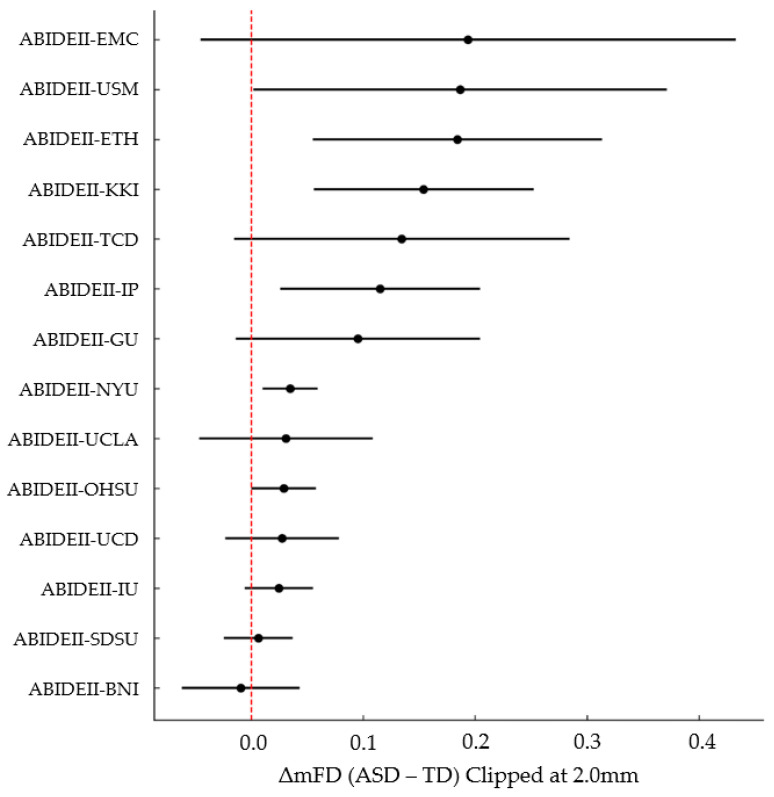
Site-wise Forest Plot of ΔmFD (ASD−TD) with ±95% C.I. Clipped at 2.0 mm.

Figure 6 summarizes the site-wise differences in mean framewise displacement (ΔmFD) between autistic and typically developing participants, illustrating both the consistency and heterogeneity of motion effects across ABIDE-II sites. Most sites show positive ΔmFD values, indicating higher motion in ASD, but the magnitude varies substantially—from near-zero differences to large site-specific effects exceeding 0.10 mm. A small number of sites show negligible or slightly negative differences, reflecting either comparable motion levels or minimal TD > ASD effects. The broad confidence intervals at some locations highlight limited precision in smaller samples, while the overall pattern reinforces that elevated motion in ASD is a dominant but heterogeneous phenomenon. This forest plot visually underscores the necessity of modeling site as a random effect and cautions against assuming uniform diagnostic differences across multi-site datasets. Distribution of mfd in participants with ASD and TD controls is shown before (left panel) and after (right panel) motion matching. Prior to matching, ASD participants exhibited higher levels of head motion compared to TD controls. Following nearest-neighbor matching, the distributions of mFD were closely aligned between groups, indicating successful balancing of head motion. This procedure reduces motion-related confounding and allows for a more equitable comparison of image quality metrics between diagnostic groups.

**Mixed-Effects Model**

**Figure 7:**
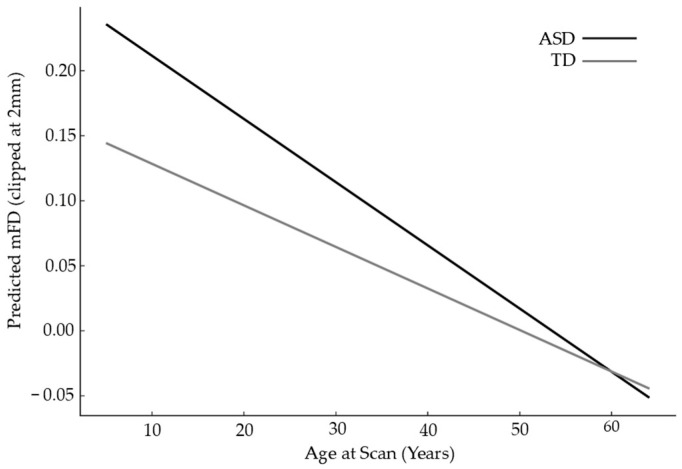
Mixed effects interaction model: age x diagnosis effect on predicted motion.

The mixed-effects model revealed that diagnostic group remained a significant predictor of head motion even after adjusting for age, sex, IQ, and site-level variability. Specifically, autistic participants exhibited an estimated 0.063 mm higher mFD than typically developing individuals, indicating consistently greater frame-to-frame head displacement across the sample. Age showed a small but significant negative association with motion, with older participants moving slightly less. In contrast, sex and IQ were not significant predictors of mFD. The inclusion of a random intercept for site demonstrated meaningful between-site variability in baseline motion levels, underscoring the necessity of modeling site effects explicitly in multi-site neuroimaging analyses, as shown in Table 6. As shown in Figure 7, the mixed-effects interaction model revealed largely parallel age-related declines in motion for ASD and TD groups, with no significant age × diagnosis interaction, indicating that the ASD–TD difference in motion remained stable across the developmental range.

The mixed-effects model revealed that site-level differences accounted for the majority of variance in SNR, with an intra-class correlation coefficient (ICC) of 0.76. This means that over three-quarters of the variability in SNR arises from between-site differences rather than individual participant characteristics, reflecting substantial heterogeneity in scanner hardware, acquisition protocols, and imaging environments across ABIDE-II sites (Table 7). The random intercept variance for Site_ID (4.514; SD = 2.124) far exceeded the residual variance (1.405), further emphasizing the dominant role of site in determining SNR.

After accounting for site effects and relevant covariates, diagnosis (ASD vs. TD) was not a significant predictor of SNR (β = −0.099, *p* = 0.221). This indicates that the modest group-level SNR difference observed in unadjusted comparisons is largely attributable to age, sex, or site-related factors, rather than to autism itself. In contrast, age and sex emerged as strong predictors of SNR. Older participants showed significantly lower SNR (β = −0.044, *p* < 0.001), consistent with known age-related changes in tissue contrast and MR signal properties. Males exhibited substantially lower SNR than females (β = 0.795, *p* < 0.001), suggesting either sex-related neuroanatomical differences or sex-distributed variation across sites that influence SNR. Full-scale IQ did not significantly predict SNR (β = 0.004, *p* = 0.128).

Sensitivity analyses excluding ASD-only sites (KUL and NYU) yielded identical results, with no change in the estimated diagnostic effect on motion. This indicates that inclusion of these sites did not bias the global model estimates.

Overall, these results demonstrate that site effects dominate SNR variance, while diagnosis does not independently influence SNR once these confounds are controlled. This underscores the importance of modeling site explicitly when analyzing multi-site neuroimaging datasets, particularly for signal-level metrics that are sensitive to scanner and protocol differences.

**Figure 8:**
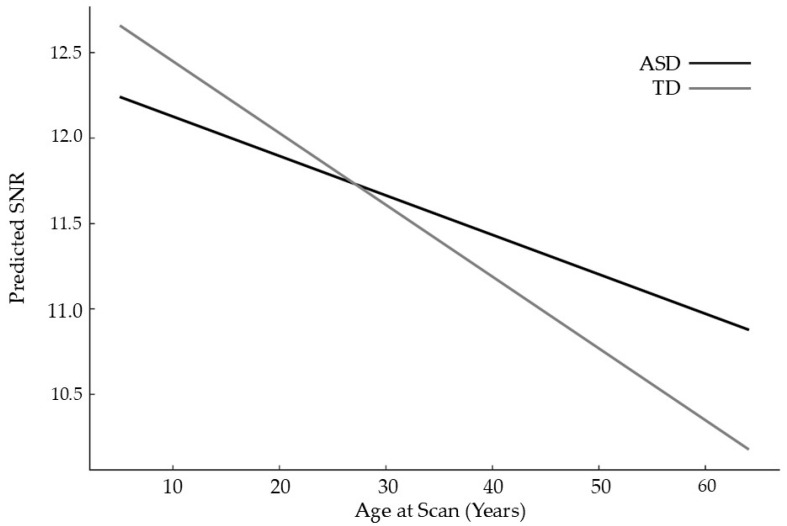
Mixed-Effects interaction model: Age Vs. Diagnosis Effect on Predicted SNR.

Figure 8 depicts the predicted age-related trajectories of signal-to-noise ratio (SNR) for autistic and typically developing participants. Both groups show a gradual decline in SNR with age, consistent with known developmental and physiological changes that influence tissue contrast and BOLD signal properties. Although the interaction term was statistically significant, the difference in slopes between groups was extremely small: the ASD trajectory declines by approximately −0.023 SNR units per year, while the TD trajectory declines by −0.042 units per year. As a result, the predicted lines remain almost entirely overlapping across the age span. This indicates that diagnostic status has minimal influence on age-related changes in SNR, and that the modest unadjusted group differences in SNR observed earlier are better explained by site effects and demographic covariates than by autism itself. Overall, the interaction model confirms that SNR trajectories are highly similar across groups and that diagnosis contributes negligibly to developmental variation in signal quality.

**Quality-control (QC) exclusion and diagnostic bias**

QC analyses revealed significant diagnostic differences in scan exclusion rates across all criteria (Table 8). Using a threshold of mFD > 0.20 mm, 21.4% of ASD participants (124/579) were excluded compared with 11.9% of TD participants (83/698), a highly significant difference (χ^2^ = 20.44, *p* = 6.1 × 10^−6^). Similarly, when applying the criterion PercentFD > 0.20 > 20%, 29.9% of ASD participants (173/579) failed QC versus 18.2% of TD participants (127/698), again yielding a significant group difference (χ^2^ = 23.39, *p* = 1.3 × 10^−6^). When combining both thresholds into a single Fail ANY criterion, 30.9% of ASD participants (179/579) were excluded compared with 18.5% of TD participants (129/698), representing the strongest effect (χ^2^ = 27.10, *p* = 3.3 × 10^−7^). These results demonstrate that ASD participants are significantly more likely to fail standard motion-based QC thresholds, indicating a robust diagnostic bias introduced by commonly used preprocessing pipelines.

**Figure 9:**
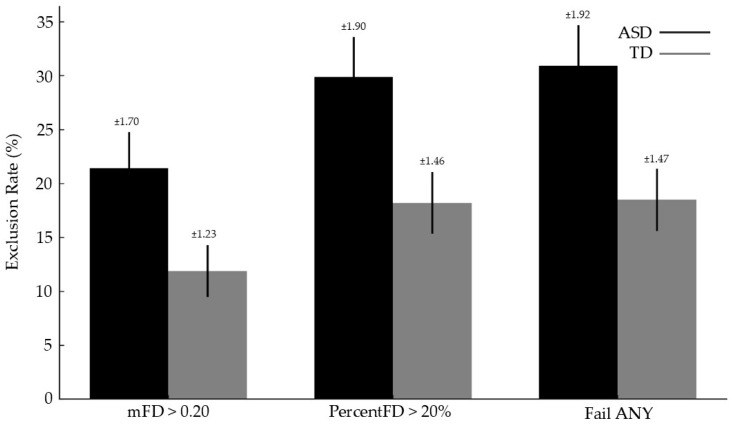
QC exclusion rates by diagnostic group (with 95% C.I.).

The QC exclusion figure visually highlights the pronounced diagnostic imbalance produced by standard motion-based filtering procedures. Across all commonly used thresholds, the bars corresponding to autistic participants are consistently higher than those for typically developing controls, demonstrating that ASD participants are substantially more likely to be removed due to excessive motion or high proportions of contaminated volumes. The divergence between groups becomes especially evident under stricter or combined exclusion rules, where ASD exclusion rates approach one-third of the sample. This visual pattern reinforces the statistical results from Table 8, showing that routine QC criteria systematically reduce ASD representation in the dataset. Such differential exclusion has important methodological implications, as it risks introducing diagnostic bias, altering sample characteristics, and potentially skewing downstream neuroimaging findings if not properly accounted for (Figure 9).

QC-related exclusion produced only modest changes in group-level phenotypic profiles (Table 9). Among ASD participants, those excluded for excessive motion showed slightly higher mean FIQ (107.56 ± 18.14) than those retained (104.23 ± 17.42), yielding a small effect size (Cohen’s d = −0.19). In the TD group, exclusion effects were minimal, with nearly identical FIQ profiles for retained (115.77 ± 12.06) and excluded participants (114.31 ± 15.05; d = 0.12). These findings indicate that QC filtering does not preferentially remove lower- or higher-functioning individuals, though it alters the ASD sample composition to a small extent.

**Figure 10:**
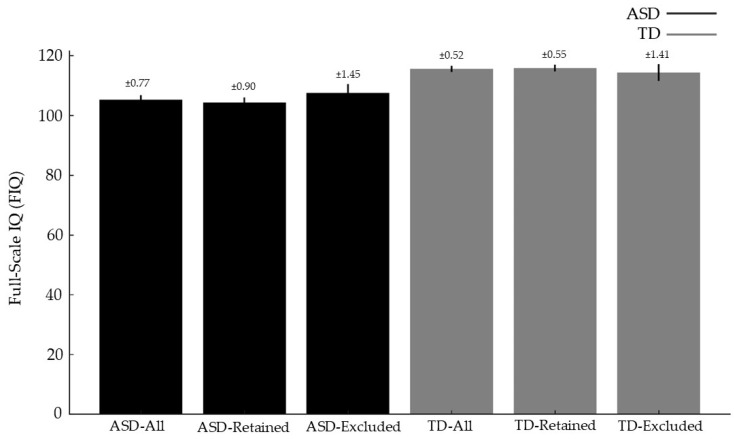
FIQ Distribution before and after AQC Exclusion (95% C.I.).

Figure 10 compares FIQ distributions before and after QC exclusion, showing that removing high-motion scans has only a minimal impact on the cognitive profile of the retained sample. In the ASD group, the post-exclusion distribution shifts slightly upward, reflecting a modest increase in mean FIQ among excluded participants; however, this effect is small and the overall shape of the distribution remains largely preserved. In the TD group, the pre- and post-exclusion FIQ distributions are nearly identical, indicating that QC filtering does not systematically favor individuals with higher or lower cognitive scores. Together, the figure illustrates that although QC exclusion disproportionately reduces the number of autistic participants, it does not meaningfully alter the cognitive composition of either diagnostic group. This suggests that motion-related data loss introduces diagnostic bias in sample size but not a strong bias in cognitive ability.

## 4. Discussion

The present study provides the most comprehensive multi-site characterization to date of diagnostic differences in MRI scan quality across ABIDE-II. Using standardized QAP metrics from 1277 participants spanning 14 sites, we demonstrate that autistic individuals exhibit consistently and substantially elevated head motion relative to TD controls across all displacement-based indices. These effects were large, robust, and highly significant, replicating and extending prior reports of increased motion in autism [4,7,8,10,11,12,13]. Importantly, these differences persisted even after adjusting for age, sex, IQ, and site-related variability in mixed-effects models, underscoring that increased movement is a stable, reproducible characteristic of autistic participants rather than an artifact of specific sites or demographic differences.

A major contribution of this study is the demonstration that motion is the dominant source of autism–control scan-quality differences, whereas other fMRI signal metrics—particularly SNR, FBER, voxel smoothness, and GCORR—showed either minimal or no diagnostic differences. Although the ASD group presented slightly lower SNR and higher EFC, these effects were small and disappeared after covariate and site adjustment. This aligns with growing evidence that many apparent group differences in MRI signal quality are driven by motion-induced artifacts rather than intrinsic neurobiological properties [10,11,14,15]. Our findings therefore highlight the distinction between motion-related artefacts and signal-level degradation: autistic participants move more, but the underlying signal properties of their scans remain largely comparable to those of TD individuals once motion and site factors are accounted for.

**Site Heterogeneity and the Importance of Multilevel Modeling**

Consistent with earlier multi-site neuroimaging work [16,17,18,19], we observed substantial heterogeneity in baseline scan quality across ABIDE-II sites. Motion levels varied nearly fourfold across centers, and the magnitude of ASD–TD differences ranged from negligible to very large. These findings reinforce that site is a major determinant of observed data quality, driven by scanner manufacturer, hardware age, pulse sequence characteristics, participant management, and institutional protocols. Our mixed-effects analyses showed that site-level variance accounted for the majority of variability in SNR and a sizable fraction of the variance in motion, emphasizing the necessity of explicitly modeling site effects when analyzing ABIDE-I/II or any other large multi-site dataset.

The fact that ASD–TD differences were consistently positive in 12 of 14 sites—despite considerable heterogeneity—demonstrates the robustness of the diagnostic motion effect. However, the magnitude of this effect is strongly site-dependent. Consequently, studies that ignore site as a random factor risk both inflated false positives and spurious group differences driven by local acquisition conditions rather than diagnostic characteristics.

**Diagnosis vs. Age Effects**

Interaction models showed largely parallel age-related declines in motion for ASD and TD individuals, with slopes nearly identical across the lifespan. This suggests that the autism-related motion difference is developmentally stable, not restricted to childhood or adolescence. For SNR, although a statistically detectable diagnosis × age effect emerged, the effect size was extremely small, and predicted trajectories remained nearly overlapping. This confirms that diagnosis has little to no influence on age-related signal-quality changes, and that the modest unadjusted group differences in SNR are largely attributable to site-level variation and demographic covariates.

**Quality Control Procedures Introduce Diagnostic Bias**

One of the most consequential findings of this work is that standard motion-based quality control procedures—widely used in preprocessing pipelines such as fMRIPrep (an automated, standardized preprocessing pipeline for fMRI data designed to maximize reproducibility and minimize user-dependent analytical choices), C-PAC (C-PAC is a highly configurable fMRI preprocessing and analysis pipeline developed by the Child Mind Institute), and Connectome-style workflows (class of preprocessing workflows optimized for functional connectivity and network analyses), systematically and disproportionately exclude autistic participants. Depending on the threshold, 21–31% of ASD scans failed QC compared with only 12–19% of TD scans. These differences were large, statistically significant, and consistent across multiple exclusion criteria.

This diagnostic imbalance poses serious methodological and interpretive risks for autism neuroimaging research. If nearly one-third of autistic participants are removed prior to analysis, resulting samples may become biased toward individuals with higher functioning levels, fewer sensory sensitivities, or greater tolerance for MRI procedures [14,19]. Although our simulations showed only modest FIQ shifts following QC exclusion, the loss of participants alone drastically reduces representativeness and statistical power, and may distort estimates of functional connectivity, cortical morphology, or developmental trajectories. Several recent studies have warned that group differences in connectivity attributed to autism may in fact reflect residual motion artefacts or QC-driven sampling biases [10,11,13,14,15]. Our findings provide direct empirical support for this concern and emphasize that QC pipelines themselves can act as a form of implicit selection bias.

DVARS (D temporal VARiance of the time series) indexes global temporal variability in the BOLD signal by quantifying the root mean square change in voxel-wise intensity between successive volumes. Unlike framewise displacement (FD), which measures physical head motion, DVARS reflects the signal consequences of motion as well as other sources of instability such as physiological or scanner-related effects. In this study, DVARS did not differ between ASD and TD participants despite clear group differences in FD, indicating that increased head displacement in ASD did not result in greater global BOLD signal instability. This dissociation supports the conclusion that ASD–TD differences are driven primarily by displacement-based motion rather than widespread temporal degradation of fMRI signal quality.

These considerations underscore the need for alternative strategies to address motion-related artefacts in neuroimaging studies. Rather than relying exclusively on exclusion-based QC thresholds, several complementary approaches may be adopted. First, incorporating motion metrics as covariates in statistical models allows for the retention of a broader and more representative sample while accounting for motion-related variance. Second, weighted or propensity-based methods may help correct for differential exclusion probabilities across groups. Third, data-driven QC approaches that treat image quality as a continuous variable, rather than dichotomizing participants into “pass” or “fail,” may offer a more nuanced and less biased framework for quality assessment. Collectively, these strategies can help balance data quality with sample representativeness.

In this context, QC procedures should be viewed not merely as a technical preprocessing step, but as a potential source of bias that can shape study conclusions. This issue is particularly relevant in longitudinal neuroimaging and biomarker research, where subtle changes over time are often interpreted as reflecting disease progression. Recent studies have emphasized the importance of accurately modeling imaging-derived biomarkers and their trajectories over extended periods [20,21]. In such settings, variability arising from head motion, site-related differences, or QC procedures may obscure or distort true biological signals, potentially affecting the identification of change points or associations with clinical outcomes. Accordingly, careful handling of image quality and motion-related artefacts is essential not only for cross-sectional analyses but also for the validity of longitudinal inferences.

**Implications for Autism Neuroimaging Research**

Taken together, our results show that motion is both the primary and the most diagnostically consequential data-quality challenge in autism MRI research. The combination of: (1) robust ASD > TD motion effects; (2) very small true signal-quality differences; (3) overwhelming site-driven variability; and (4) strong QC-induced diagnostic bias creates a methodological landscape in which interpretation of group differences is particularly fragile. These findings underscore several key recommendations for future studies:

1. Motion should not be treated solely as a nuisance covariate, but as a primary scientific variable whose distribution and group differences must be transparently reported.
2. Site effects must be explicitly modeled using mixed-effects models or harmonization approaches such as ComBat or hierarchical Bayesian frameworks.
3. QC thresholds should be re-evaluated, potentially adopting adaptive or diagnosis-aware criteria, and analyses should report both pre- and post-QC sample characteristics.
4. Motion-mitigation strategies—mock scanner training, prospective motion correction, real-time feedback, and quieter sequences—should be prioritized when studying populations with sensory and behavioral variability.
5. Data-sharing initiatives should include richer metadata on acquisition context (e.g., whether participants were sedated, trained, or scanned with real-time motion tracking) to enable more nuanced modeling of heterogeneity.

Our findings align strongly with a growing literature showing that autistic participants exhibit higher motion [4,7,8,10,11,12,13], that site contributes substantially to variability in MRI quality [16,17,18,19,20,21,22], and that QC procedures have the potential to introduce diagnostic bias [14,15,22]. Although QC thresholds are not universally standardized, the thresholds used in this study reflect commonly adopted practices in the field. Therefore, the observed diagnostic differences in exclusion rates are likely to reflect typical preprocessing conditions rather than artefacts of an arbitrary cutoff choice. However, this study extends prior work by incorporating the full ABIDE-II QAP dataset, applying consistent mixed-effects modeling, quantifying ASD–TD differences across a wider set of quality metrics, and explicitly evaluating the downstream consequences of QC exclusion on sample composition. As such, it provides one of the clearest demonstrations to date that methodological factors, not neural biology per se, are responsible for many apparent group differences in autism MRI studies.

**Limitations**

This study has several limitations that should be considered when interpreting the results. First, although ABIDE-II provides one of the largest multi-site autism neuroimaging datasets, its heterogeneity remains substantial. Scanner hardware, acquisition protocols, and site-specific preprocessing procedures introduce variability that cannot be fully accounted for, even with linear mixed-effects models and random intercepts. Second, the analysis focused primarily on fMRI quality metrics derived from the ABIDE-II QAP outputs; structural MRI quality was not examined, and therefore the findings may not generalize to all imaging modalities. Third, because the dataset is observational and cross-sectional, causal inferences, such as whether autism intrinsically contributes to poorer scan quality, cannot be firmly established.

Differences in motion may reflect diagnostic characteristics, sensory sensitivities, anxiety, age distributions, or contextual differences in site protocols, none of which can be disentangled completely using available metadata. Another limitation concerns the use of standard QC thresholds. Although we quantified how QC procedures disproportionately exclude autistic participants, the thresholds themselves are not universally standardized across the field.

Different preprocessing pipelines (e.g., ICA-AROMA, fMRIPrep, censoring strategies) may produce different exclusion profiles, limiting the direct generalizability of our simulations. Additionally, QAP metrics capture only certain aspects of signal quality and may be insensitive to physiological noise sources such as respiration, heart rate, or local susceptibility artifacts, which could differ between groups or sites but are not measured here. Finally, although mixed-effects modeling allowed us to estimate diagnosis, age, and site contributions simultaneously, the available sample sizes for individual sites, especially those contributing only ASD participants, limit the precision of site-specific estimates. Site-level analyses should therefore be interpreted cautiously, particularly for sites with small sample sizes, as limited statistical power may affect the precision and stability of site-specific estimates. In addition, substantial heterogeneity was observed across acquisition sites in both head motion and image quality metrics, likely reflecting differences in scanner hardware, acquisition protocols, and sample characteristics. Although site was modeled as a random effect in the mixed-effects analyses, residual variability may persist, and site-level estimates should therefore be interpreted with caution, particularly for sites with smaller sample sizes. The lack of complete age and IQ matching across sites also restricts the extent to which demographic confounds can be fully addressed. Future work using harmonized acquisition protocols, richer phenotypic metadata, and longitudinal designs will be essential to validate and extend the present findings.

**Practical Recommendations for Improving MRI Compliance in ASD**

Although methodological adjustments and statistical controls can mitigate the impact of motion on MRI data, an equally important avenue—often underemphasized in multi-site neuroimaging studies—is the implementation of participant-centered strategies that reduce motion at the source. Given the robust and pervasive ASD–TD differences in displacement metrics observed in this study, it is essential to consider environmental, procedural, and educational adaptations that can enhance autistic individuals’ comfort and cooperation during MRI scanning. A growing body of research demonstrates that such strategies can meaningfully reduce in-scanner motion and improve data yield without compromising standard imaging protocols [20,21,22,23,24,25].

One of the most consistently supported interventions is mock scanner training, in which participants undergo a simulated MRI session to familiarize themselves with scanner noise, confined space, and required stillness. Structured mock-scan desensitization has been shown to dramatically increase successful scan completion in autistic children, with improvements persisting across multiple sessions [20]. Similarly, behavioral rehearsal protocols—including step-by-step modeling, reinforcement, and gradual exposure—have been shown to reduce motion and increase scan success [8]. These approaches help participants build predictable expectations and reduce anxiety-driven restlessness, addressing two of the most common sources of movement during MRI.

In addition to preparatory training, sensory and communication accommodations within the scanning environment play a crucial role in improving compliance. Autistic individuals often experience heightened sensitivities to noise, brightness, and tactile stimuli, and these sensory factors can exacerbate motion. Simple modifications, including dimmable lighting, comfortable padding, weighted blankets, or personalized noise-dampening solutions, can increase tolerability and reduce behavioral distress [25]. Visual schedules, clear stepwise instructions, and advanced explanations of the scanning process have likewise been reported to enhance cooperation, particularly for participants with difficulties in verbal communication or high levels of uncertainty.

Technological innovations also offer promising pathways for reducing motion in ASD populations. Real-time motion feedback systems and prospective motion correction techniques allow participants or technicians to monitor and adjust motion during acquisition. Providing visual feedback about head movement has been shown to significantly reduce displacement in autistic adolescents and adults [23]. These approaches align closely with the needs of ASD participants, who often benefit from explicit, immediate reinforcement and clear performance cues. Moreover, naturalistic stimulus paradigms—such as watching movies or meaningful social scenes—reduce restlessness and support longer periods of stillness [24].

Finally, scan protocols themselves may be optimized to meet the needs of neurodivergent populations. Shorter scanning blocks, scheduled breaks between runs, fast-acquisition sequences, and minimizing transitions between tasks can all reduce accumulated motion and participant fatigue. Creating ASD-friendly MRI rooms, characterized by predictable routines, reduced sensory load, and staff trained in neurodiversity-informed communication, represents a low-cost but high-impact strategy that imaging centers can adopt. These modifications do not require psychological intervention or clinical therapy; rather, they constitute evidence-based procedural enhancements that directly address the elevated motion patterns documented in this study.

Together, these practical strategies underscore the broader methodological implication of our findings: improving data quality in autism neuroimaging requires not only statistical solutions but also participant-centered adaptations grounded in sensory and behavioral science. Incorporating these recommendations into routine imaging practice may reduce diagnostic bias introduced by QC exclusion, increase data retention in ASD groups, and improve the validity and generalizability of multi-site MRI research.

## 5. Conclusions

Across ABIDE-II sites, autistic participants showed consistently higher head motion, while signal and spatial quality differences were minimal, indicating that motion is the primary diagnostic disparity in fMRI data quality. Standard QC thresholds disproportionately excluded autistic individuals, introducing systematic diagnostic bias without meaningfully altering cognitive profiles. Site effects accounted for substantial variance, underscoring the need for site-aware modeling. Together, these findings highlight motion, site heterogeneity, and QC bias as critical methodological factors that must be addressed to ensure valid and reproducible autism neuroimaging results.

## Figures and Tables

**Table 1:** ABIDE II Site Identifiers and Full Institution Names.

Site ID	Full Institution Name
**ABIDEII-BNI**	Barrow Neurological Institute (BNI)
**ABIDEII-EMC**	Erasmus University Medical Center (EMC), Rotterdam
**ABIDEII-ETH**	ETH Zürich (Swiss Federal Institute of Technology)
**ABIDEII-GU**	Georgetown University (GU)
**ABIDEII-IP**	Institut Pasteur (IP), Paris
**ABIDEII-IU**	Indiana University (IU)
**ABIDEII-KKI**	Kennedy Krieger Institute (KKI), Baltimore
**ABIDEII-NYU**	New York University (NYU) Child Study Center
**ABIDEII-OHSU**	Oregon Health & Science University (OHSU)
**ABIDEII-SDSU**	San Diego State University (SDSU)
**ABIDEII-TCD**	Trinity College Dublin (TCD)
**ABIDEII-UCD**	University of California, Davis (UCD)
**ABIDEII-UCLA**	University of California, Los Angeles (UCLA)
**ABIDEII-USM**	University of Southern Mississippi (USM)

**Table 2:** MRI quality metrics derived from the ABIDE-II Quality Assessment Protocol (QAP), grouped into motion-related and signal/spatial domains.

Metric Name	Abbreviation	Description
**Motion-related**		
**Mean Framewise Displacement**	mFD	Average instantaneous head displacement between successive volumes across the scan; primary indicator of overall head motion.
**Percent of Volumes with FD > 0.20 mm**	PercentFD greater_than_0.20	Percentage of volumes exceeding the 0.20 mm framewise displacement threshold, reflecting frequency of excessive motion.
**Number of Volumes with FD > 0.20 mm**	NumFD greater_than_0.20	Absolute count of high-motion volumes, complementing the percentage-based metric.
**Outlier Count**	OutlierCount	Proportion of volumes flagged as statistical outliers due to abnormal voxel-wise signal deviations.
**Median Distance Index**	MDI	Median distance between each volume and a reference volume, indexing temporal signal instability related to motion.
**D temporal VARiance of the time series**	DVARS	Included as a complementary metric reflecting frame-to-frame changes in BOLD signal intensity, providing an index of global temporal signal variability associated with motion and other sources of instability.
**Signal/Spatial**		
**Signal-to-Noise Ratio**	SNR	Ratio of anatomical signal strength to background noise; higher values indicate cleaner and more reliable scans.
**Entropy Focus Criterion**	EFC	Entropy-based measure of image sharpness and ghosting; higher values indicate increased blurring or reduced focus.
**Spatial Smoothness**	FWHM (Smoothness of Voxels)	Full-width at half maximum of spatial autocorrelation, estimating effective image smoothness and resolution.
**Foreground-to-Background Energy Ratio**	FBER	Ratio of mean energy inside the brain to that in non-brain regions, reflecting contrast quality.
**Global Correlation**	GCORR	Average voxel-wise correlation across the brain, indexing widespread signal dependencies and potential artifacts.

**Table 3:** Global ASD vs. TD Differences in Motion-Related QAP Metrics (fMRI).

Metric	ASD (n = 579)	TD (n = 698)	*p*-Value	Interpretation
**mFD**	0.101 mm (0.06–0.17)	0.081 mm (0.05–0.13)	1.1 × 10^−10^	ASD shows significantly higher head motion
**Percent FD > 0.20 mm**	8.84% (1.97–23.23)	4.31% (0.78–13.84)	4.0 × 10^−10^	ASD has a higher proportion of high-movement volumes
**Num FD > 0.20 mm**	13 (3–36)	6 (1–23)	1.1 × 10^−10^	ASD produces more high-movement frames
**Outlier** **Fraction**	0.004 (0.00–0.01)	0.002 (0.00–0.01)	2.9 × 10^−10^	ASD scans contain more outlier volumes
**DVARS**	1.14 (1.07–1.24)	1.15 (1.07–1.25)	0.57	No significant ASD–TD difference

**Table 4:** Global ASD vs. TD Differences in Signal and Spatial QAP Metrics (fMRI).

Metric	ASD (n = 579)	TD (n = 698)	*p*-Value	Interpretation
**SNR**	12.66 (9.92–15.27)	13.41 (10.82–15.62)	2.8 × 10^−4^	TD exhibits modestly higher SNR; ASD shows slightly noisier signal
**EFC**	0.452 (0.405–0.512)	0.430 (0.392–0.492)	8.1 × 10^−11^	ASD scans show higher EFC, indicating less optimal focus/entropy
**FBER**	189.4 (153.7–241.5)	191.8 (158.3–244.9)	0.27	No significant group difference in foreground-to-background energy
**FWHM**	4.91 (4.45–5.41)	4.87 (4.42–5.36)	0.38	Spatial smoothness did not differ significantly between groups
**GCORR**	0.074 (0.058–0.093)	0.072 (0.057–0.089)	0.11	Global correlation similar across groups
**MDI**	0.062 (0.053–0.073)	0.060 (0.052–0.070)	0.055	Trend-level higher values in ASD, not statistically significant

SNR: Signal-to-Noise Ratio; EFC: Entropy Focus Criterion; FBER: Foreground-to-Background Energy Ratio; FWHM: Spatial Smoothness; GCORR: Global Correlation; MDI: Median Distance Index.

**Table 5:** Site-wise mFD differences for sites with both ASD and TD participants.

Site_ID	N_ASD	N_TD	mFD_ASD (mm)	mFD_TD (mm)	ΔmFD (ASD–TD)	S.E.	95% C.I.
**ABIDEII-BNI_1**	29	29	0.156	0.156	0.000	0.03	0.05
**ABIDEII-EMC_1**	27	27	0.123	0.153	–0.030	0.12	0.23
**ABIDEII-ETH_1**	13	24	0.203	0.096	0.107	0.06	0.12
**ABIDEII-GU_1**	51	55	0.128	0.093	0.035	0.05	0.10
**ABIDEII-IP_1**	64	98	0.088	0.038	0.049	0.04	0.08
**ABIDEII-IU_1**	20	20	0.063	0.063	–0.000	0.01	0.03
**ABIDEII-KKI_1**	56	155	0.165	0.103	0.061	0.05	0.09
**ABIDEII-NYU_1**	48	30	0.097	0.055	0.042	0.01	0.02
**ABIDEII-OHSU_1**	111	168	0.100	0.078	0.022	0.01	0.02
**ABIDEII-SDSU_1**	33	25	0.060	0.056	0.004	0.01	0.03
**ABIDEII-TCD_1**	21	21	0.152	0.088	0.064	0.07	0.14
**ABIDEII-UCD_1**	18	14	0.082	0.067	0.015	0.02	0.05
**ABIDEII-UCLA_1**	16	16	0.113	0.101	0.011	0.03	0.07
**ABIDEII-USM_1**	17	16	0.170	0.078	0.092	0.09	0.18

**Table 6:** Linear Mixed-Effects Model Predicting Mean Framewise Displacement (mFD). Model includes Site_ID as a random intercept. Dependent variable: clipped mFD (values > 2 mm truncated). N = 1120 participants.

Predictor	Estimate (β)	SE	Test Statistic (z)	*p*-Value	Interpretation
**Intercept**	0.142	0.020	7.10	<0.001	Baseline mFD for reference participant (TD, female, mean age, mean FIQ)
**Diagnosis** **(ASD vs. TD)**	0.063	0.012	5.03	<0.001	ASD participants show significantly higher mFD after adjusting for covariates
**Age at Scan** **(years)**	−0.003	0.001	−2.38	0.018	Older participants move slightly less
**Sex**	0.003	0.012	0.24	0.81	No meaningful sex difference in motion
**Full-Scale IQ** **(FIQ)**	0.0004	0.0003	1.07	0.29	IQ does not significantly predict motion
**Random Effect: Site_ID**	0.003	—	—	—	Substantial site-level variability in baseline motion

**Table 7:** Linear Mixed-Effects Model Predicting Signal-to-Noise Ratio (SNR). Model includes Site_ID as a random intercept. Dependent variable: SNR. N ≈ 1120 participants.

Predictor	Estimate (β)	SE	Test Statistic (z)	*p*-Value	Interpretation
**Intercept**	12.915	0.633	20.391	<0.001	Baseline SNR for reference participant (TD, female, mean age, mean FIQ)
**Diagnosis (ASD vs. TD)**	−0.099	0.08	−1.225	0.221	After covariate adjustment, ASD does not differ significantly from TD in SNR
**Age at Scan (years)**	−0.044	0.007	−5.876	<0.001	Older participants tend to have lower SNR
**Sex**	−0.795	0.087	−9.148	<0.001	Males show significantly lower SNR than females
**Full-Scale IQ (FIQ)**	0.004	0.002	1.523	0.128	IQ shows a small, non-significant positive association with SNR
Random Effect Component	**Site_ID variance**	**Site_ID SD**	**Residual variance**	**Residual SD**	**ICC (site-level)**
	4.514	2.124	1.405	1.186	0.763

**Table 8:** χ^2^ test Quality-Control (QC) Exclusion Rates and Diagnostic Bias (fMRI). QC failure defined using thresholds: mFD > 0.20 mm OR PercentFD > 0.20 > 20%.

QC Criterion	ASD Fail	ASD %	TD Fail	TD %	χ^2^	*p*-Value	Interpretation
**mFD > 0.20 mm**	124/579	21.4%	83/698	11.9%	20.44	6.1 × 10^−6^	ASD participants are almost twice as likely to exceed the motion threshold
**PercentFD > 0.20 > 20%**	173/579	29.9%	127/698	18.2%	23.39	1.3 × 10^−6^	ASD participants produce significantly more high-motion frames
**Fail ANY criterion**	179/579	30.9%	129/698	18.5%	27.10	3.3 × 10^−7^	QC exclusion disproportionately removes ASD participants

“Fail ANY” row best represents real-world QC exclusion, since most pipelines use combined criteria.

**Table 9:** Change in Phenotypic Profile (FIQ) After QC Exclusion. C failure defined as mFD > 0.20 mm OR PercentFD > 0.20 > 20%.

Group	Mean FIQ (All)	SD	Mean FIQ (Retained)	SD	Mean FIQ (Excluded)	SD	Cohen’s d	Interpretation
**ASD**	105.22	17.69	104.23	17.42	107.56	18.14	**−0.19**	Excluded ASD participants have slightly higher FIQ; effect is small
**TD**	115.49	12.69	115.77	12.06	114.31	15.05	**+0.12**	TD exclusion changes FIQ minimally (very small effect)

## Data Availability

The data analyzed in this study are publicly available through the Autism Brain Imaging Data Exchange II (ABIDE-II) initiative. fMRI quality metrics (Quality Assessment Protocol; QAP) and phenotypic datasets were obtained from the ABIDE-II repository, accessible via the International Neuroimaging Data-sharing Initiative (INDI) platform (http://fcon_1000.projects.nitrc.org/indi/abide/abide_II.html, accessed on 27 January 2026). Access to ABIDE-II data requires registration and agreement to the data usage terms established by the data contributors. No new raw imaging data were generated as part of this study. All analyses were conducted using previously released, de-identified datasets. Derived analysis code and statistical scripts used to generate the results reported in this manuscript are available from the corresponding author upon reasonable request.

## Abbreviations

The following abbreviations are used in this manuscript:

**Abbreviation**	**Full Term**	**Description**
**ABIDE**	Autism Brain Imaging Data Exchange	Multi-site open database of MRI and phenotypic data for autism research.
**ABIDE II**	Autism Brain Imaging Data Exchange II	Second phase of ABIDE with additional sites and subjects.
**AQC**	Automated Quality Control	Automated detection/exclusion of low-quality scans based on predefined thresholds.
**ASD**	Autism Spectrum Disorder	Neurodevelopmental condition; primary clinical group in the study.
**BOLD**	Blood Oxygen Level–Dependent	fMRI signal contrast based on blood oxygenation.
**CI**	Confidence Interval	Interval estimate reflecting uncertainty around a parameter or prediction.
**DVARS**	D temporal VARiance of Time Series	Measure of frame-to-frame intensity changes in the BOLD signal.
**DX**	Diagnosis	General term for diagnostic classification (ASD vs. TD).
**DX_GROUP**	Diagnostic Group	Phenotypic variable coding diagnostic status (1 = ASD, 2 = TD).
**EFC**	Entropy Focus Criterion	Entropy-based metric reflecting image sharpness and ghosting.
**FD**	Framewise Displacement	Instantaneous head motion between consecutive volumes.
**FWHM**	Full-Width at Half Maximum	Estimate of spatial smoothness or effective resolution of the image.
**FBER**	Foreground-to-Background Energy Ratio	Ratio of mean energy inside the brain to that in the background.
**FIQ**	Full-Scale Intelligence Quotient	Global cognitive ability score used as a covariate.
**GCORR**	Global Correlation	Average voxel-wise correlation across the brain, indicating global signal dependencies.
**ICC**	Intraclass Correlation Coefficient	Proportion of total variance attributable to between-site differences.
**ID**	Identifier	Generic subject or record identifier.
**LMM**	Linear Mixed-Effects Model	Regression model including both fixed and random effects.
**MRI**	Magnetic Resonance Imaging	Neuroimaging technique used to acquire structural and/or functional brain images.
**QAP**	Quality Assessment Protocol	Set of automated metrics used to quantify MRI data quality.
**QC**	Quality Control	Procedures used to evaluate and filter imaging data based on quality.
**IQ**	Intelligence Quotient	General measure of cognitive ability; includes FIQ and subscales.
**IQR**	Interquartile Range	Measure of statistical dispersion between the 25th and 75th percentiles.
**mFD**	Mean Framewise Displacement	Average framewise displacement across the entire scan.
**MedianDistanceIndex**	Median Distance Index	Median distance between each volume and a reference volume, reflecting temporal instability.
**NumFD** **greater_than_0.20**	Number of Volumes with FD > 0.20 mm	Absolute count of timepoints exceeding the 0.20 mm threshold.
**OutlierCount**	Outlier Count	Proportion or count of volumes flagged as statistical outliers.
**PercentFD** **greater_than_0.20**	Percent of Volumes with FD > 0.20 mm	Percentage of timepoints exceeding the 0.20 mm displacement threshold.
**TD**	Typically Developing	Control group without autism diagnosis.
**SE**	Standard Error	Standard error of an estimate, often used in plotting uncertainty bands.
**Site_ID**	Site Identifier	Code indicating the acquisition site or data collection center.
**SNR**	Signal-to-Noise Ratio	Ratio of signal intensity to noise; core indicator of image clarity.
**χ^2^**	Chi-Squared	Statistical test used to compare observed vs. expected frequencies.
